# Optimizing Backfill Materials for Ground Heat Exchangers: A Study on Recycled Concrete Aggregate and Fly Ash for Enhanced Thermal Conductivity

**DOI:** 10.3390/ma17235876

**Published:** 2024-11-30

**Authors:** Andrzej Głuchowski

**Affiliations:** Institute of Civil Engineering, Warsaw University of Life Sciences—SGGW, 166 Nowoursynowska Str., 02787 Warsaw, Poland; andrzej_gluchowski@sggw.edu.pl; Tel.: +48-22-5935405

**Keywords:** thermal conductivity, industrial solid wastes, construction and demolition materials, thermal needle probe, moisture content, compaction

## Abstract

This study investigates the potential use of recycled concrete aggregate (RCA), fly ash (FA), and their mixture (RCA+FA) as backfill materials for shallow vertical ground heat exchangers (GHEs). Granulometric, aerometric, and Proctor compaction tests were conducted to determine soil gradation, the void ratio, and the optimal moisture content (OMC) for maximum dry density. RCA demonstrated efficient compaction at lower moisture levels, while FA required higher moisture to reach maximum density. A 10% FA addition was optimized to fill voids in the RCA soil skeleton without compromising structural stability. Thermal conductivity tests were performed using a TP08 probe in both dry and wet states. The results showed that the RCA+FA mix exhibited a notable increase in thermal conductivity at around 6% moisture content due to the formation of water bridges between particle contacts. FA, in contrast, displayed a more linear relationship between conductivity and moisture. The RCA+FA mix achieved higher thermal conductivity than either material alone, particularly near full saturation, making it a promising option for efficient heat exchange. Thermal conductivity modeling, based on the Woodside and Messmer model, confirmed the RCA+FA mix’s high conductivity and estimated full saturation conductivity values with a small error. The Kersten number (*K_e_*) was employed to predict conductivity across varying moisture levels, with results showing a strong correlation with saturation ratio (*S_r_*).

## 1. Introduction

Shallow geothermal energy, provided by a ground source heat pump (GSHP) systems, has proven efficient for both cooling and heating buildings [1,2]. The advantage of GSHP with ground heat exchangers (GHEs) lies in their cost-effectiveness and energy-efficient performance. GHEs, located just a few meters below the surface, can act as heat sources in winter and heat sinks in summer [3,4]. Designing GHEs requires detailed information about the soil’s thermal properties, particularly its thermal conductivity (λ). Soil moisture conditions in the unsaturated layer lead to high variability in λ, requiring special attention during the design process [5]. Shallow GHEs are often in a non-fully saturated state, which can significantly reduce thermal conductivity. Even small changes in moisture content can impact the coefficient of performance (COP) of the GSHP [6,7]. System effectiveness increase can be achieved by enhancing the thermal conductivity of the GHE backfill material [8,9]. For this purpose, the backfill must achieve thermal conductivity that is higher than, or at least equal to, that of the grouts [10]. For instance, vertical boreholes, commonly filled with bentonite or a bentonite–cement mix, exhibit effective thermal conductivity levels between 0.65 and 0.90 W/mK under saturated conditions [11].

In unsaturated soils, heat conduction depends heavily on the soil’s thermal conductivity, as heat transfer occurs mainly through the soil skeleton, and water content, which enhances contact between soil particles [12]. The key aspect impacting thermal conductivity is the physical soil conditions in which the GSHP operates: (i) soil dry density (*ρ_d_*), (ii) moisture content (*m*) or the saturation ratio (*S_r_*), (iii) porosity (*n*), (iv) mineralogy or origin, and (v) water retention capabilities.

Water content affects both soil–water suction stress and the state of thermal conductivity. Water drains from the pores as saturation decreases, and meniscus water wets only the particle contacts. In a dry state, only adsorbed water remains [13]. Soil minerals have thermal conductivity values that vary, ranging from 1.8 W/m·K for illite, 2.8 W/m·K for kaolinite, and 3.4 to 7.8 W/m·K for quartz [14]. Saturated sand has a thermal conductivity between 1.7 and 2.5 W/m·K, while clayey soils have values between 0.94 and 1.29 W/m·K, which is more than four times and twice as high as the thermal conductivity of water (0.594 W/m·K), respectively, under the best-case scenario [15,16]. One simple method for increasing soil thermal conductivity, aside from raising moisture content, is compaction. Reducing the void volume increases the number of connections between particles, leading to greater thermal conductivity. However, compaction may not achieve the desired results for poorly graded soils like sand. To overcome this, adding fines to the sand mixture can improve soil thermal properties [17]. Another way to enhance soil thermal conductivity is to stabilize soil with cementitious solutions, which fill the pores and bond soil skeleton particles. This method has a twofold effect: it reduces the void ratio by filling the pores and strengthens the bonds between soil particles [18,19]. To ensure proper shallow GHE performance, it is essential to maintain sufficient soil-heat conductivity, even in low-moisture conditions. 

Non-cohesive soils with high gradation have a low field capacity, meaning they retain less pore water through adhesion as the pore volume increases, and they can remain fully saturated only when the groundwater table is above the soil layer. When the groundwater level drops, free pore water infiltrates due to gravitational forces [20]. One possible solution to this problem is to increase soil gradation distribution to achieve a well-graded particle size distribution. However, such soils pose challenges due to their low permeability and expansive nature [21,22]. Solid waste materials can serve as alternatives to natural soils, offering a wide range of properties that differ from those of natural soils with similar gradation. These materials often exhibit unique behaviors compared to their natural counterparts. Commonly used solid waste materials in civil engineering include industrial solid waste (ISW) and construction and demolition (C&D) materials [23,24].

Fly ash (FA) is an example of ISW that has a wide range of applications. FA, being coal combustion residue, constitutes over 70% of waste coal ash [25]. The FA utilization rate varies from 90% in Europe to 50% for the US and 67% for China, but the world average is 25% [26]. FA is classified into Class C and Class F, depending on its chemical composition, with Class F containing more than 70% SiO_2_, Al_2_O_3_, and Fe_2_O_3_, and Class C having 50–70% [27]. Class C FA has a higher calcium content (above 15% CaO) and self-cementing properties. In contrast, Class F fly ash, with less than 5% CaO, possesses pozzolanic properties but requires an activator to form cement [28]. The geotechnical properties of FA may vary, depending on the FA origin and combustion conditions. The hydraulic conductivity is in the range of 1·10^−3^ to 1·10^−7^ cm/s, and the internal friction angle, φ’, between 23 to 43°, indicating the need for prior laboratory testing [25].

A widely used construction and demolition (C&D) material in geotechnical engineering is recycled concrete aggregate (RCA). This material often surpasses its natural counterparts in terms of strength and overall bearing capacity [24,29,30,31]. RCA, being a byproduct of concrete crushing, consists of soil grains and mortar, with the mortar thickness depending on the RCA grain diameter. Strength tests indicate that lower-density RCA experiences more grain crushing, leading to a reduction in strength. Therefore, proper compaction is crucial for achieving high strength [32]. RCA has been successfully applied as a base and sub-base material for pavements [33,34].

This article presents the thermal conductivity properties of fly ash (FA) and RCA blends. The primary goal is to propose an alternative to natural aggregates and soils for vertical GHE fill. The FA–RCA mix was chosen to combine the high strength of RCA with the favorable properties of FA, such as high water retention and non-swelling characteristics. This mix can be used as a heat transfer medium for vertical GHEs, reducing the overall resistivity of the heat exchange system.

## 2. Materials and Methods

### 2.1. Material

**Figure 1:**
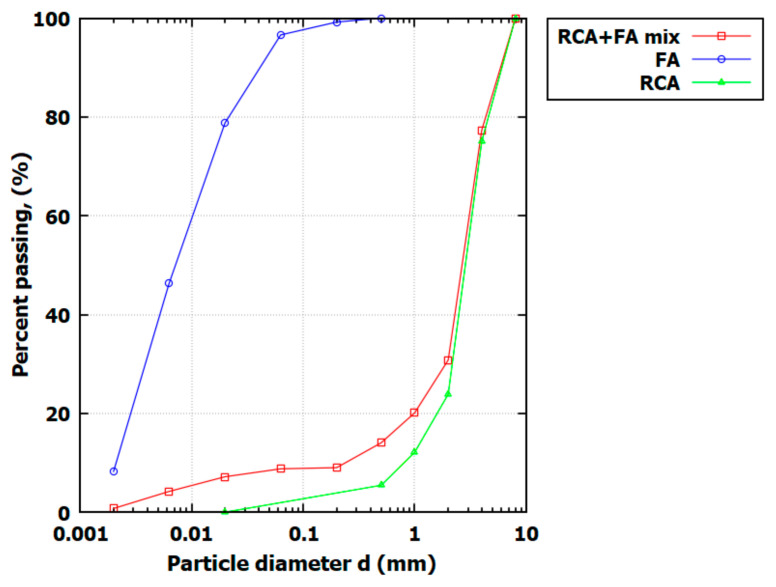
Soil gradation curves for FA, RCA, and RCA+FA mix.

For FA, RCA, and the RCA+FA mix, granulometric and aerometric tests were conducted to determine soil gradation composition. The grain size distribution is presented in Figure 1. The RCA gradation ranged from 0.05 to 8 mm, with the 2–4 mm fraction being dominant. The coefficient of curvature (CC) and coefficient of uniformity (CU) for RCA, shown in Table 1, indicate that RCA is poorly graded, and based on the USCS classification, it can be categorized as poorly graded gravel with sand (GP). FA gradation, ranging from 0.5 mm to 0.0 mm, had a dominant fraction between 0.02 mm and 0.0063 mm, with CU and CC values indicating a poorly graded material. This soil type can be classified as low-plasticity silt (ML).

Tests for minimum and maximum void ratios were conducted for FA and RCA to determine the optimal FA addition to RCA. The optimal addition should be small enough not to disrupt the RCA soil skeleton connections between grains yet large enough to fill the voids in the RCA skeleton, allowing the RCA+FA mix to benefit from FA’s high water absorption properties. Based on the test results shown in Table 1, the FA addition was set to 10%.

FA’s effect after it is mixed with RCA led to a decrease in the *e_min_* from 0.552 to 0.204. The void ratio measures the volume of voids in a soil sample relative to the volume of solid soil particles. Therefore, the decrease in the *e_min_* to 0.204 shows that voids in compacted soil are smaller than for virgin RCA. This leads to higher water retention capabilities and higher RCA+FA-mix field capacity. The field capacity, *F_C_*, refers to the amount of pore water that soil can retain after excess water has drained away due to gravity to the volume of pores. In this study, field capacity was measured after the soil had been saturated (saturation ratio *S_r_* = 1) and allowed to drain freely for a period (usually 24–48 h). The results are presented in Table 1.

**Figure 2:**
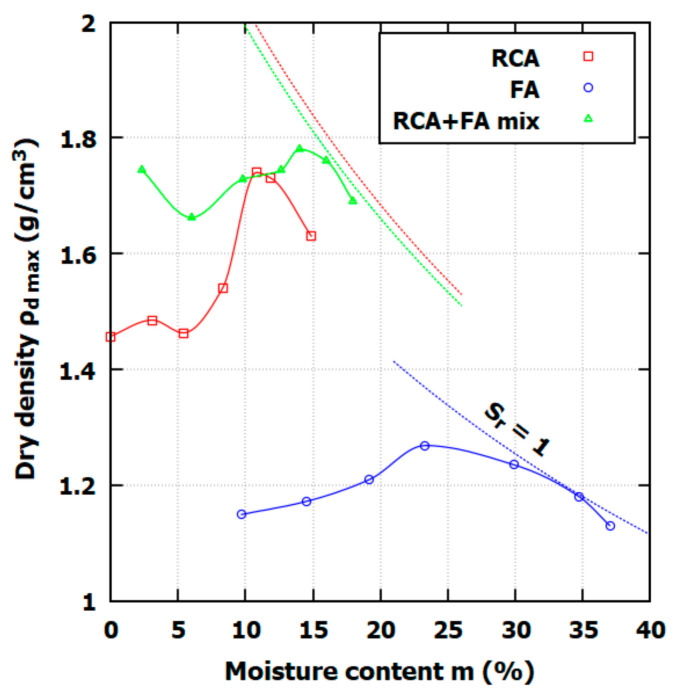
Results of the Proctor test for RCA, FA, and RCA+FA mix. The dotted lines presented full saturation conditions.

Shallow vertical GHE for a higher heat exchange and layer stability is compacted in the optimal moisture content (OMC) to obtain the maximum dry density (*ρ_d max_*). Therefore, the Proctor compaction test was carried out in this study to determine both parameters. The laboratory test was performed with respect to ASTM recommendations using the standard Proctor test, in which the compaction energy is equal to 593.7 kJ/m^3^. The results of the Proctor test are presented in Figure 2. In Table 2, the OMC and dry density for soils tested in this study are presented.

The results of the Proctor tests offer a few insights regarding soil-heat thermal properties: (i) RCA compaction characteristics show a rapid dry density increase close to OMC and a later rapid *ρ_d max_* drop; (ii) FA presents as typical for this material type compaction curve, where OMC occurs at a high moisture content; (iii) FA achieves a full saturation ratio relatively easily in contrast to RCA, for which full saturation conditions during compaction are not possible; and (iv) the RCA+FA mix combines both material properties, the overall density is high, reaching 1.781 g/cm^3^, and the moisture content reaches almost full saturation during compaction.

### 2.2. Thermal Conductivity Measurement

A conductivity sensor denoted as TP08 (Huskeflux, Delft, The Netherlands) was used in this study. TP08 is a non-steady-state probe (NSSP) that relies on the transient line heat source measurement method also known as the thermal needle or hot wire technique. The probe consists of a heading wire, which is a perfect line heat source, temperature sensors measuring heat source temperature, and environment regions. The procedure of the test covers (i) sample preparation and compaction, (ii) sensor installation and thermal conductivity measurement, and (iii) post-test physical properties’ measurement.

#### 2.2.1. Sample Preparation and Compaction

The sample preparation phase involved placing the FA+RCA mix in a cylinder with a 0.15 m diameter and 0.15 m height. The compaction procedure was conducted with respect to the normal Proctor energy of compaction equal to 0.59 J/cm^3^ and a 2.5 kg hammer. The compaction was conducted in 3 layers to achieve uniform density across the sample. During the third layer compaction, when 80% of the blow numbers were finished, the excess soil was removed from the cylinder’s top, and the remaining 20% of the required number of hammer blows was conducted.

#### 2.2.2. Sensor Installation and Thermal Conductivity Measurement

**Figure 3:**
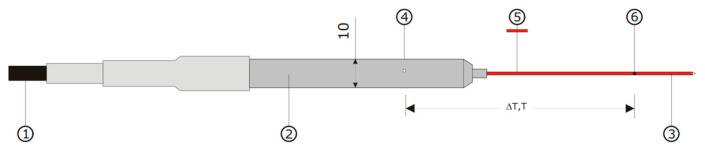
The TP08 probe consists of a needle (3) with a single thermocouple junction (6) and a heating wire (5). It is inserted into the medium that is investigated. In the base, (2), a temperature sensor, (4), is mounted [35].

The TP08 sensor has a diameter of 1.2 mm and a length of 70 mm, and it is made from stainless steel instrumented with temperature sensors (thermocouple and thermistors) and heating wire to supply thermal energy. The TP08 sensor is presented in Figure 3. The sensor type, in which the heating wire is a source of heat, represents a perfect linear heat source. 

The TP08 operates in the temperature range of 218.15 to 453.15 K with an accuracy of ±3%. The TP08 probe was calibrated before the tests using glycerol with standard thermal conductivity (*λ* = 0.286 W/mK), for which the calibration constant, C, was calculated as follows (1):

(1) $$C=\frac{\lambda_{material}}{\lambda_{measured}},$$

The NSSP principle relies on a unique property of a line source: after a short transient period, usually 100 to 200 s, the temperature rise, ∆*T*, depends only on the heater power, *Q*, and the medium thermal conductivity, *λ* (2):

(2) $$∆T=\frac{Q\cdot \ln\;t\cdot C}{4\pi \cdot \lambda },$$

Equation (1) can be redrawn, so thermal conductivity, λ, is calculated as follows (3):

(3) $$\lambda =\frac{CQ\cdot \ln\;t}{4\pi \cdot ∆T}=\frac{CQ}{4\pi \cdot m},$$

where the following applies: *Q* is the power supply value (W), *m* is the average linear gradient of the linear part of the curve (*m* = ln(*t*)/∆T), ∆*T* is the temperature gradient (K), and *t*—is time (s).

**Figure 4:**
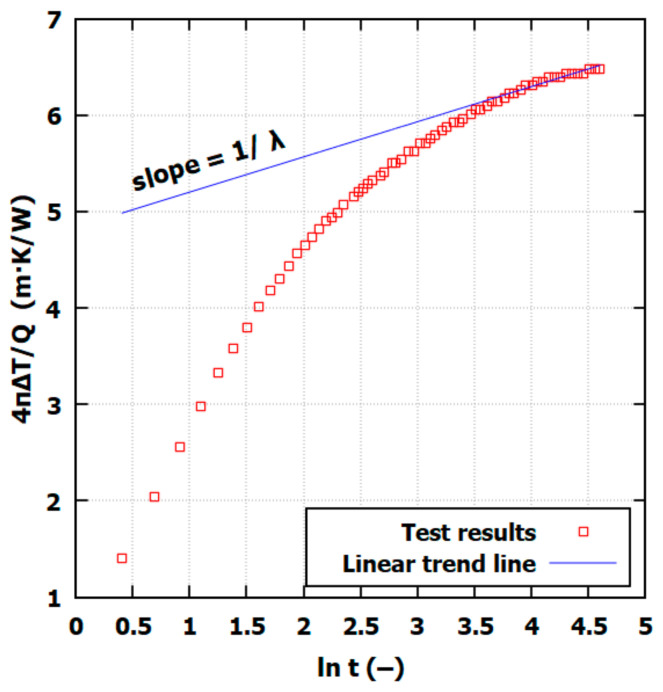
Typical measurement of thermal conductivity measurement with NSSP with *λ* estimation.

Following the standard ASTM method, the heating phase duration should be at least 60 s to ensure test accuracy. The heating phase was set to 100 s. The first 30 to 60 s were ignored to ensure the measurement accuracy. The linear part of the curve presented in Figure 4 must be fitted to the rest of the time interval for a thermal conductivity calculation. The measurements of *Q*, *t*, and ∆*T* are all direct measurements of power, time, and temperature, respectively.

The sensor was installed in the sample in the center of the cylinder so that the whole needle was placed in the sample (according to the manufacturer’s recommendation, at least 20 mm of the needle should be embedded in the sample).

#### 2.2.3. Post-Test Physical Properties’ Measurement

After thermal conductivity was measured, the sample underwent a series of physical-property tests to determine the saturation ratio (*S_r_*), dry density (*ρ_d_*), and void ratio (*e*). These properties were calculated based on each sample’s mass and moisture content.

## 3. Results

### 3.1. Thermal Conductivity Test Results

Thermal conductivity tests were conducted for RCA, FA, and the RCA+FA mix in terms of density and moisture content. The goal of the tests was to test soil in a wide range of moisture contents, especially in the dry and wet states. The results of the thermal conductivity test are presented in Table 3.

**Figure 5:**
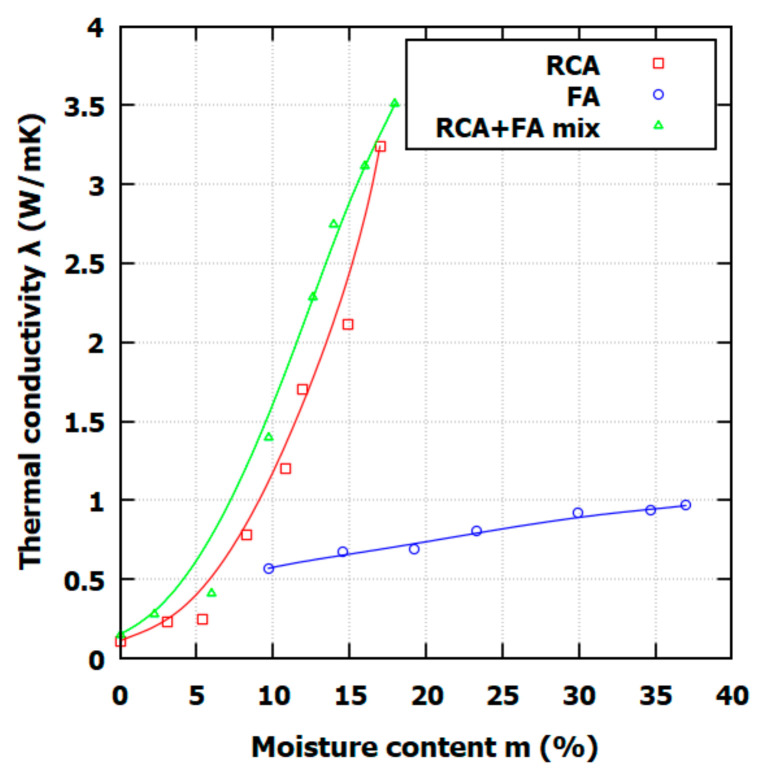
Relationship between thermal conductivity and moisture content for FA, RCA, and RCA+FA mix.

**Figure 6:**
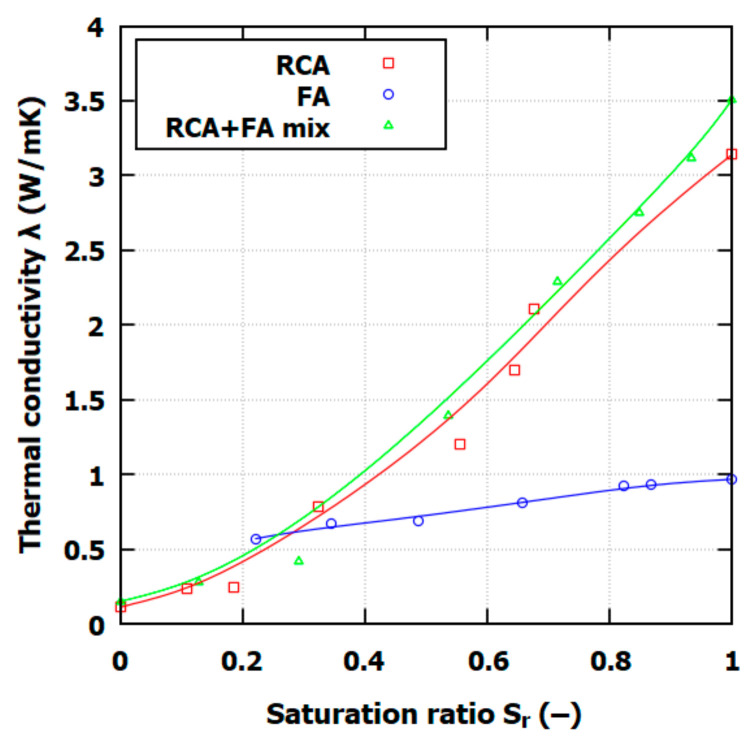
Relationship between thermal conductivity and saturation ratio *S_r_* for FA, RCA, and RCA+FA mix.

The function of moisture content is presented in Figure 5. What is worth noting is that, in the case of RCA and the RCA+FA mix, soils exhibit a rapid change in thermal conductivity when the moisture content reaches a specific value. The water content is too small for both soils in a dry state to create so-called *water bridges* between the particle contacts. Contact surfaces between particles often have small areas, so the heat conduction greatly depends on pore water enclosing the contacts. The threshold moisture content responsible for the creation of these water bridges is around 6% for the RCA and RCA+FA mix. For FA, such phenomena were not observed. The reason is that the FA gradation is small enough to assure high particle contacts and that FA is composed of glass–amorphous silica with *λ_S_*~1.1 W/mK. Since moisture content relates water mass to solid mass, to study the effect of pore water on water bridges forming, the saturation ratio, *S_r_* (defined as the volume of water, *V_w_*, to the volume of pores, *V_p_*) is more useful. The relationship between *λ* and *S_r_* is presented in Figure 6.

The saturation ratio value can range from 0 to 1; *S_r_* = 0 represents the dry state, and *S_r_* = 1 represents full saturation state. For all soils tested in this study, *λ* increases with *S_r_*. When *S_r_* is low, the surface of soil particles is covered by only a thin layer of water film, which has almost no effect on *λ.* The *λ*(*S_r_*) dependency for FA is close to linear characteristics, but the RCA and RCA+FA mix shows a nonlinear relationship, meaning that the water film appears at higher *S_r_* levels. The thermal conductivity characteristics are closely related to the RCA up to *S_r_* = 0.4. When *S_r_* is higher than 0.7, *λ* increases, surpassing the RCA thermal conductivity characteristics. 

### 3.2. Thermal Conductivity Modeling

To utilize the test results, the Woodside and Messmer model for calculating thermal conductivity was used to calculate thermal conductivity for full-saturation conditions *λ_sat_* [36]. The model is described as follows (4) and (5):

(4) $$\lambda_{sat}={\lambda_{s}}^{1-n}{\lambda_{w}}^{n},$$

(5) $$\lambda_{s}={\lambda_{q}}^{q}{\lambda_{0}}^{1-q},$$

where *n* is soil porosity (the ratio of the volume of voids, *V_v_*, to the soil volume, *V*), $\lambda_{w}$ (0.594 W/m K) is the water’s thermal conductivity, $\lambda_{0}$ is taken as 2.0 W/mK for soils with *q* > 0.2, 3.0 W/mK for soils with *q* ≤ 0.2, *q* is the quartz content in the soil, and $\lambda_{q}$ (7.7 W/mK) is the thermal conductivity of quartz [37].

The quartz content was set to 0.85 for RCA since the number of aggregates oscillated around this percentage. For FA, since the amorphous silica is the main component, the $\lambda_{0}$, it was decided, was set to 1.1 W/mK, representing glass thermal conductivity, and the amount of quartz was set to be equal to 0. Table 4 presents the results of the calculations.

For all three soils, the error in the $\lambda_{sat}$ calculation was less than 10%. Overall, the Woodside and Messmer model underestimates the $\lambda_{sat}$ value, but the error occurs at an acceptable range.

Modeling of thermal conductivity for different saturation ratios requires the use of a formula that utilizes the Kersten number, *K_e_*, and the normalized thermal conductivity [38], presented in Equations (6) and (7):

(6) $$K_{e}=\left(\lambda_{eff}-\lambda_{dry}\right)/\left(\lambda_{sat}-\lambda_{dry}\right),$$

(7) $$\lambda_{eff}=K_{e}\left(\lambda_{sat}-\lambda_{dry}\right)+\lambda_{dry},$$

where $\lambda_{dry}$ is the thermal conductivity of dry soil (*S_r_* = 0). The normalized thermal conductivity model assumes that soil thermal conductivity is a linear combination of soil thermal conductivities when the soil is dry ($\lambda_{dry}$) and saturated ($\lambda_{sat}$). The results of the Kersten number calculation for soils in this study are presented in Table 5.

**Figure 7:**
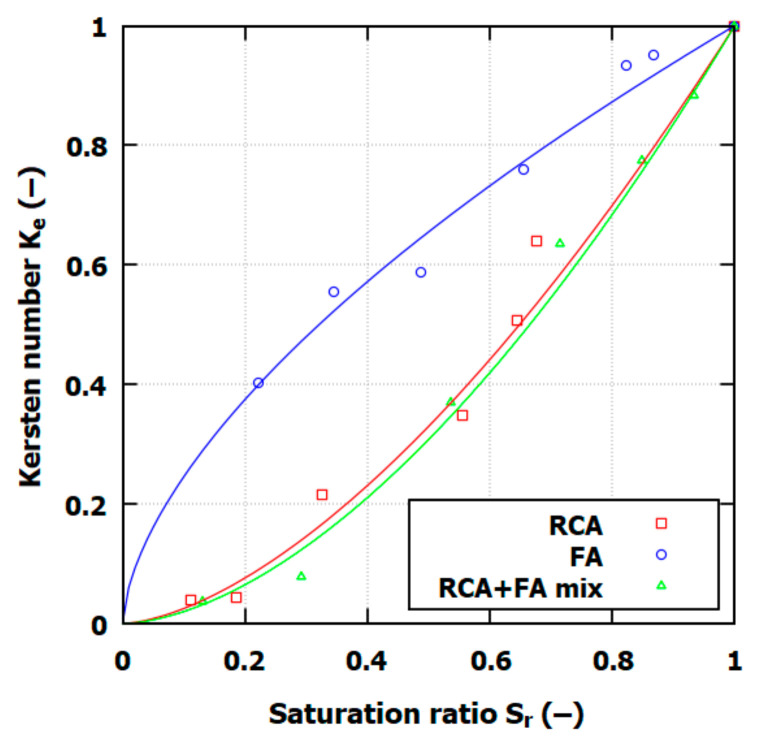
Kersten number and saturation ratio characteristics from thermal conductivity tests (points) with fitted power function (lines).

The Kersten number shows a strong relationship with the *S_r_* value, and for the thermal conductivity modeling of the soils in this study, a power relationship between *K_e_* and *S_r_* was developed as follows (8):

(8) $$K_{e}={S_{r}}^{k},$$

where *k* is the material constant equal to 1.6, 0.61, and 1.7 for RCA, FA, and the RCA+FA, mix respectively. The relationship between the Kersten number and the saturation ratio is presented in Figure 7. For the fitted power function, the coefficient of determination, R^2^, was higher than 0.978.

The Kersten number value follows previous observations regarding moisture content. For RCA and RCA+FA, the *K_e_* increase rate is higher for *S_r_* > 0.6; for FA, rather the opposite characteristic is observed, and for *S_r_* > 0.6, the *K_e_* increase rate is smaller.

## 4. Discussion

The thermal conductivity results for RCA, FA, and the RCA+FA mixes align with those from prior studies on recycled and composite backfill materials. The RCA and RCA+FA mixtures show an initial low thermal conductivity in dry states, followed by a significant increase in thermal conductivity at approximately a medium moisture content, which is the effect of water bridges’ formation between particles. This observation agrees with the literature findings, in which the same shift in the *λ* value is observed around *S_r_* = 0.4 [39]. This behavior contrasts with FA, exhibiting a more linear increase in thermal conductivity with moisture content, consistent with previous observations of fly ash or bentonite–fly ash backfills that showed similar linear conductivity changes [40,41].

For FA, the thermal conductivity plateau effect is observed in the literature as well. This effect can be observed when fines are forming high capillary forces [16]. This effect, however, was not observed in RCA and RCA+FA due to the large particle size and varied porosity distribution, which supports a stronger moisture-dependent response.

The literature suggests that the thermal conductivity of FA alone is typically lower, which tends to increase the volumetric air content and lower conductivity values [42]. This study’s results support this, with FA having relatively low thermal conductivity compared to RCA, particularly in unsaturated conditions; additionally, the results highlight the potential of FA as an insulating material under certain GHE conditions. In contrast, RCA and RCA+FA mixtures achieved much higher conductivities as saturation approached full levels, a trend consistent with studies on recycled concrete (RC) mixtures. In comparison, the thermal conductivity of various waste materials at full saturation can vary widely. For example, crushed brick has *λ_sat_* = 1.197 W/mK, a sand–rubber mix has *λ_sat_* = 2.2 W/mK, and autoclaved aerated concrete has *λ_sat_* = 0.935 W/mK. The RCA+FA mix exhibits higher thermal conductivity than these materials; however, the testing conditions should be carefully considered for accurate comparisons [39,43].

The Woodside and Messmer model and Kersten number (*K_e_*) evaluation for various saturation levels have shown that all three soils in this study demonstrated a nonlinear increase in thermal conductivity with saturation. The same findings for FA and RCA can be found in the literature, where increased water content enhances conductivity to a point, after which changes are minimal, suggesting a saturation threshold for effective thermal transfer [44,45].

Before applying the RCA+FA mix as a backfill for GHEs, environmental impact studies are essential due to potential leachate risks. Both fly ash and RCA can release harmful trace elements, especially in acidic conditions, which could lead to groundwater contamination [46,47].

## 5. Conclusions

This study investigated the thermal conductivity and compaction properties of recycled concrete aggregate (RCA), fly ash (FA), and their mix (RCA+FA mix) to evaluate their suitability as backfill materials for shallow vertical ground heat exchangers (GHEs). The findings highlight RCA+FA’s potential as a cost-effective and sustainable alternative for GHE applications. RCA compacts effectively at lower moisture levels (OMC = 10.8% and *ρ_d max_* = 1.738 g/cm^3^) compared to FA, which requires higher moisture (OMC = 23.3%) for maximum density (*ρ_d max_* = 1.268 g/cm^3^). The RCA+FA mix exhibited the highest dry density (OMC = 13.9% and *ρ_d max_* = 1.781 g/cm^3^), offering stable and dense backfill properties ideal for efficient heat transfer. The thermal conductivity (λ) of all materials increased with the moisture content, with RCA and RCA+FA showing a nonlinear response due to water-bridge formation at around 6% moisture content, while FA exhibited a linear trend. These results underscore the RCA+FA mix’s potential to optimize conductivity under high-saturation conditions. The thermal conductivity in the full saturation state for FA, RCA, and the RCA+FA mix is equal to 0.967, 3.24, and, 3.51 W/mK, respectively.

To explore the thermal conductivity modeling, a validation of the predictive model of the Woodside and Messmer for full saturation and the Kersten number (*K_e_*) for varying moisture levels was conducted. For FA modeling, $\lambda_{0}$, it was decided, was set to 1.1 W/mK to reflect amorphous silica mineral composition. The Kersten number shows a strong correlation with the saturation ratio (*S_r_*), with a power relationship developed for the studied material constant of k = 1.6 for RCA, 0.61 for FA, and 1.7 for RCA+FA, achieving a coefficient of determination (R^2^) that exceeded 0.978.

The findings confirm that the RCA+FA mix can effectively combine favorable thermal and compaction characteristics, offering a sustainable solution to backfill design. To extend this study, further tests in field conditions including long-term moisture retention and environmental interactions need to be conducted. Exploring alternative compositions of waste material could further enhance the versatility of sustainable backfill solutions in thermal and geotechnical engineering projects.

## Figures and Tables

**Table 1:** Physical properties of soils tested in this study.

Property	FA	RCA	RCA+FA
CU	4.2	15.5	3.56
CC	0.61	6.13	1.73
*e_max_*	1.008	0.976	0.907
*e_min_*	0.178	0.552	0.204
G_S_	2.31	2.54	2.52
*F_C_*	0.92	0.46	0.83

**Table 2:** Proctor test results—OMC and dry density for materials tested in this study.

Property	FA	RCA	RCA+FA
OMC	23.3	10.8	13.9
*ρ_d max_*	1.268	1.738	1.781

**Table 3:** Results of thermal conductivity test for soils in this study.

*m* (%)	*ρ_d_* (g/cm^3^)	*n* (−)	*S_r_* (−)	*λ* (W/mK)
**RCA**				
0	1.46	0.425	0.00	0.110
3.1	1.49	0.413	0.11	0.233
5.4	1.46	0.425	0.19	0.244
8.3	1.54	0.394	0.32	0.782
10.8	1.74	0.315	0.56	1.2
11.9	1.73	0.319	0.65	1.7
14.9	1.63	0.358	0.68	2.11
17.4	1.6	0.370	1.00	3.24
**FA**				
9.7	1.15	0.428	0.22	0.569
14.5	1.17	0.417	0.35	0.671
19.2	1.21	0.398	0.49	0.692
23.3	1.27	0.369	0.66	0.807
29.9	1.25	0.376	0.82	0.923
34.7	1.20	0.403	0.87	0.934
37.0	1.13	0.438	1.00	0.967
**RCA+FA mix**				
0.0	1.64	0.349	0.00	0.15
2.3	1.71	0.321	0.13	0.278
6.0	1.66	0.340	0.29	0.415
9.8	1.73	0.314	0.54	1.396
12.6	1.74	0.308	0.71	2.286
14.5	1.78	0.294	0.85	2.75
16.1	1.76	0.302	0.93	3.12
18.7	1.69	0.329	1.00	3.51

**Table 4:** Woodside and Messemer thermal conductivity model calculation results.

Soil	q	$\lambda_{0}$	n	$\lambda_{satEq(4)}$	$\lambda_{satTEST}$
RCA	0.85	2.0	0.319	2.96	3.24
FA	0.0	1.1	0.403	0.86	0.93
RCA+FA mix	0.765	2.0	0.253	3.17	3.51

**Table 5:** Kersten number calculation based on Equation (6) for thermal conductivity in this study.

RCA		FA		RCA+FA mix	
*S_r_*	*K_e_*	*S_r_*	*K_e_*	*S_r_*	*K_e_*
0.111	0.039	0.222	0.403	0.130	0.038
0.186	0.043	0.346	0.556	0.292	0.079
0.325	0.215	0.487	0.588	0.536	0.371
0.556	0.348	0.656	0.760	0.714	0.636
0.645	0.508	0.822	0.934	0.849	0.774
0.677	0.639	0.867	0.951	0.934	0.884
1.000	1.000	1.000	1.000	1.000	1.000

## Data Availability

Dataset available on request from the author.
